# Aviation-Related Wildland Firefighter Fatalities — United States, 2000–2013

**DOI:** 10.15585/mmwr.mm6429a4

**Published:** 2015-07-31

**Authors:** Corey R. Butler, Mary B. O’Connor, Jennifer M. Lincoln

**Affiliations:** 1 National Institute for Occupational Safety and Health, Western States Office, CDC; 2 Alaska Pacific Office, National Institute for Occupational Safety and Health, CDC

Airplanes and helicopters are integral to the management and suppression of wildfires, often operating in high-risk, low-altitude environments. To update data on aviation-related wildland firefighting fatalities, identify risk factors, and make recommendations for improved safety, CDC’s National Institute for Occupational Safety and Health (NIOSH) analyzed reports from multiple data sources for the period 2000–2013. Among 298 wildland firefighter fatalities identified during 2000–2013, 78 (26.2%) were aviation-related occupational fatalities that occurred during 41 separate events involving 42 aircraft. Aircraft crashes accounted for 38 events. Pilots, copilots, and flight engineers represented 53 (68%) of the aviation-related fatalities. The leading causes of fatal aircraft crashes were engine, structure, or component failure (24%); pilot loss of control (24%); failure to maintain clearance from terrain, water, or objects (20%); and hazardous weather (15%). To reduce fatalities from aviation-related wildland firefighting activities, stringent safety guidelines need to be followed during all phases of firefighting, including training exercises. Crew resource management techniques, which use all available resources, information, equipment, and personnel to achieve safe and efficient flight operations, can be applied to firefighting operations.

Airplanes and helicopters play a major role in the control of wildland (forest, brush, and grass) fires. These aircraft are used to deliver equipment and supplies, deploy and transport firefighters, conduct reconnaissance, scout and direct operations, and deliver fire retardant or water. During the past decade, the United States has experienced an increase in the size, frequency, and severity of wildfires, likely attributable to buildup of flammable vegetation, decline in snowpack, and human development in the wildland urban interface (1,2). If these conditions continue, more fire response workers will be needed, and the demand on aviation to support these efforts will increase.

To identify risk factors for aviation-related wildland firefighter activities, NIOSH reviewed and extracted case reports from the Fire Administration Firefighter Fatality surveillance system, the National Fire Protection Association Fire Incident Data Organization database, the National Wildland Coordinating Group’s Safety Gram, and the National Transportation Safety Board aviation database. A wildland firefighter fatality was defined as any death that occurred in a paid or unpaid wildland firefighter, contractor, aviation crew member or support staff, inmate, or member of the military while performing official wildland fire duties, including operations (fire or nonfire incident), responding to or returning from a wildland fire incident, or other officially assigned duties.* Other emergency response workers who were fatally injured at wildfires were excluded from this analysis. The number of flight hours for the U.S. Forest Service was used as a denominator to indicate the use of aviation resources because flight hours from other agencies or workforce numbers were not available.

During 2000–2013, a total of 298 wildland firefighter fatalities were identified, averaging 21 fatalities per year. Among these, 78 (26.2%) were caused by activities associated with aviation. The number of aviation- related fatalities decreased during 2007–2013, compared with 2000–2006 (Table 1). Of the persons who died in aviation-related activities, 76 (97%) were male, and 53 (68%) were flight crew members (e.g., pilots, copilots, and flight engineers). The average age of flight crew victims was 49 years (range = 20–66 years) and of nonflight crew victims was 33 years (range = 19–54 years). The most common occupation of nonflight crew members was firefighter. Most victims were employed by aerial contractors (42), followed by the federal government (15), state government agencies (10), ground contractors (seven), and the military (four). Twenty-five (32%) of the aviation-related fatalities occurred in California, eight occurred in Nevada, and seven in Idaho (Figure).

For pilots in command who were victims, the mean total flight hours (available for 34) was 10,725 hours. Most fatalities (67.5%) occurred during June–September; 31% occurred in August. Fifty-two (67%) deaths occurred during the direct support of wildland fire incidents, 12 (16%) during training exercises, and three (4%) at prescribed fires (fires that are deliberately ignited to reduce fuels, control competing vegetation, improve accessibility, and preserve forest ecology). The remaining 11 (14%) fatalities occurred during other nonemergency activities such as repositioning operations, nonemergency staffing replacements, nonfire recovery missions, or traveling to other events.

The 78 deaths occurred during 41 separate events involving 42 firefighting aircraft; 23 (55%) aircraft were fixed wing, and 19 (45%) were helicopters. Three firefighting aircraft were involved in midair collisions. Two air tankers (airplanes equipped with tanks for carrying and dumping water or retardant) collided, and a helicopter evacuating a firefighter collided with a nonfire medical evacuation helicopter while approaching a hospital.† Aircraft crashes accounted for 38 (93%) fatal events involving 75 (96%) fatalities. The remaining fatalities occurred in three separate events, two during smokejumper operations and another during rappelling operations. Twenty events involved multiple (range = two to nine) wildland firefighter fatalities. Ten (24%) fatal aircraft crashes resulted from structure or component failure, 10 (24%) from pilot loss of control; eight (20%) from failure to maintain clearance from terrain, water or objects; and six (15%) from encounters with hazardous weather (Table 2). Seven (37%) helicopters were operating with external loads when they crashed.

To increase safety, agencies have instituted policies, protocols and training requirements, including improving aircraft inspection and maintenance programs, limiting retardant loads, shifting firefighting strategies to reduce reliance on air tankers during large fires, and ending leases on some retired military aircraft that were considered high risk to firefighter safety (3). These changes have resulted in a decreased number and rate of fatalities in recent years; however, working with and around aircraft is still one of the highest risk activities for firefighters.

## Discussion

Airplanes and helicopters are commonly used in wildland fire operations to deploy and transport workers and equipment to and from a fire, apply retardant and water, perform reconnaissance of fires, and ignite prescribed fires (4). Most operations performed by aircraft involved in wildland firefighting are considered emergency response operations, and many are considered to be hazardous, such as low-level reconnaissance and water and retardant drops. During 2002–2013, more than 72,000 wildfires burned an average of 7 million acres each year; California reported the largest annual average number of fires (7,998) (5). In 2012, federal land management agencies of the U.S. Department of Agriculture and U.S. Department of the Interior, spent approximately $2 billion suppressing wildfires (6). During 1991–2012, the proportion of the Forest Service budget dedicated to controlling wildfires increased from approximately 13% to 40% (7). As the frequency, complexity, and area burned by wildfires continues to increase, the number and types of personnel and the high demand for the limited number of available aircraft to aid in wildfire suppression will continue to grow (8).

Fighting wildfires often requires a multifaceted approach, involving federal, state, tribal, and local agencies, all with different missions, legal responsibilities and authorities (9). While some agencies own their own aircraft, others rely on contracts to provide the fleet necessary for managing wildfires. Training programs, policies, individual qualifications, experience levels, crew abilities and knowledge, resources, and support structure vary among the different agencies that often work together during these hazardous operations. Using the most stringent safety guidelines available for each activity will ensure the highest level of protection for all workers. Crew resource management comprises a range of skills, knowledge, behaviors, and actions that can be applied to firefighters and firefighting operations. The Forest Service, U.S. Department of the Interior, and other agencies have incorporated crew resource management system elements (e.g., situational awareness, communication, decision-making, and risk management) that are specifically applicable to wildland firefighting safety. Incorporating such an approach to all firefighting operations might lead to increased efficiency, effectiveness, and safety.

The findings in this report are subject to at least three limitations. First, aviation-related fatalities might have occurred that were not included in the databases, especially if the incident did not occur during a wildland fire event (e.g., during maintenance, training, or in transit to a fire). Second, occupational fatality rates based on the number of wildland firefighters or the number of flight hours logged by wildland firefighters using aircraft as part of their jobs could not be calculated because those data were not available. Finally, reliable wildland firefighter workforce population estimates, including the number of aviation contractors and nonfederal flight crew members, were not available. The wildland firefighting workforce comprises mostly seasonal, volunteer, or contract workers from a variety of different employing agencies. No single data system tracks the number of wildland firefighters, and there is also no occupational code specifically for wildland firefighting.

Wildland firefighting necessitates an interagency approach, requiring many persons to work together in a complex and often unpredictable and hazardous environment. To reduce fatalities from aviation-related wildland firefighting activities, the most stringent safety guidelines need to be followed during all phases of firefighting to help firefighters, flight crews, and fire managers assess risk, limit exposure, share information, and enhance teamwork when using aircraft to control wildfires.

**Summary**

What is already known on this topic?

Working in and around aircraft is considered one of the most dangerous operations when suppressing wildfires. Aviation-related incidents are one of the leading causes of death among wildland firefighters. Many fire management agencies have instituted policies and protocols that are designed to reduce the risk associated with using aircraft when suppressing wildfires.

What is added by this report?

During 2000–2013, a total of 78 firefighters were fatally injured while performing wildland fire duties involving aircraft during 41 separate events. The leading causes of fatal aircraft crashes were engine, structure, or component failure; loss of control of the aircraft; failure to maintain clearance from terrain, water, or objects; and hazardous weather.

What are the implications for public health practice?

Agencies and employers need to ensure that the most stringent and consistent safety guidelines and standards that incorporate a comprehensive approach to efficient, effective, and safer wildland firefighting operations are followed during firefighting operations.

## Figures and Tables

**FIGURE:**
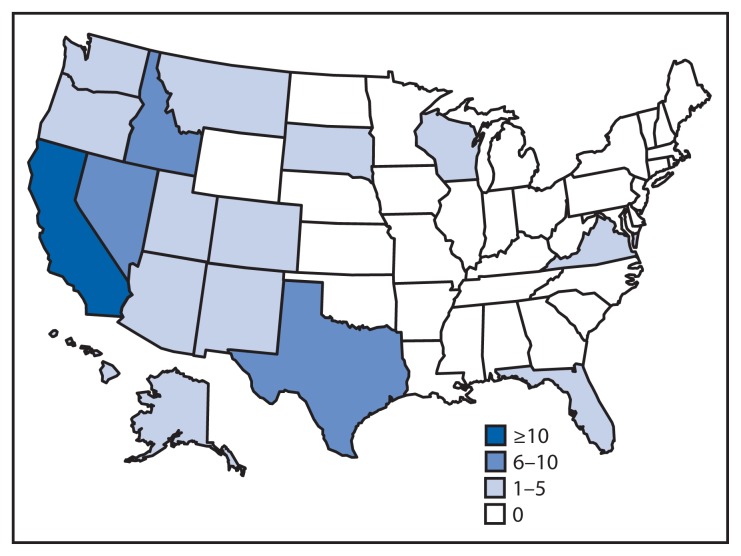
Number (N = 78) of aviation-related wildland firefighter fatalities — United States, 2000–2013 **Sources:** U.S. Fire Administration Firefighter Fatality surveillance system, National Fire Protection Association Fire Incident Data Organization data system, National Wildland Coordinating Group’s Risk Management Committee Safety Gram, Fatalities, Entrapments and Serious Accident data system, and National Transportation Safety Board aviation database.

**TABLE 1:** Fatal aviation events and associated wildland firefighter fatalities — United States, 2000–2013

	7-year interval	
	
Fatal aviation event	2000–2006	2007–2013
No. of events involving a fatality	28	13
Rate*	4.6	2.1
Average per year	4	2
No. of fatalities	49	29
Rate*	8.0	4.7
Average per year	7	4

**Sources:** U.S. Fire Administration Firefighter Fatality surveillance system, National Fire Protection Association Fire Incident Data Organization data system, National Wildland Coordinating Group’s Risk Management Committee Safety Gram, Fatalities, Entrapments and Serious Accident data system, and National Transportation Safety Board aviation database.

* Rate of fatalities per 100,000 U.S. Forest Service reported flight hours for aircraft owned, leased, or contracted by the Forest Service by fiscal year. Fiscal year was paired with calendar year to match the summer flying season (i.e., fiscal year 2000 is shown with data from calendar year 2000).

**TABLE 2:** Causes of fatal aviation events in wildland firefighting activities — United States, 2000–2013

Cause	Events	(%)	Deaths	(%)
Failure of engine, structure, or component	10	(24)	18	(23)
Loss of control (including failure to maintain airspeed)	10	(24)	15	(19)
Failure to maintain clearance from terrain, water, or obstacles	8	(20)	15	(19)
Weather	6	(15)	13	(17)
Midair collisions	2	(5)	3	(4)
Failure of parachute or rappel equipment	3	(7)	3	(4)
Weight and balance	1	(2)	9	(12)
Cause not reported	1	(2)	2	(3)
**Total**	**41**	**—** *	**78**	**—** *

**Sources:** U.S. Fire Administration Firefighter Fatality surveillance system, National Fire Protection Association Fire Incident Data Organization data system, National Wildland Coordinating Group’s Risk Management Committee Safety Gram, Fatalities, Entrapments and Serious Accident data system, and National Transportation Safety Board aviation database.

* Total does not sum to 100% because of rounding.

## Footnotes

* Wildland firefighting duties include training, reconnaissance, maintenance, public education, inspection, investigations, court testimony, or fundraising; being on call, under orders, or on standby duty, except at the individual’s home or other place of business. Additional information available at http://www.gpo.gov/fdsys/pkg/BILLS-108s459enr/pdf/BILLS-108s459enr.pdf.

† The nonfire medical evacuation helicopter with six other nonfirefighting personnel who were fatally injured during this event were not included in this analysis because they were not associated with wildland firefighting operations.
